# Co-Amoxiclav Induced Immune Haemolytic Anaemia: A Case Report

**DOI:** 10.1155/2020/9841097

**Published:** 2020-03-31

**Authors:** H. G. C. S. Karunathilaka, D. P. Chandrasiri, P. Ranasinghe, V. Ratnamalala, A. H. N. Fernando

**Affiliations:** ^1^National Hospital of Sri Lanka, Colombo, Sri Lanka; ^2^Department of Pharmacology, Faculty of Medicine, University of Colombo, Colombo, Sri Lanka

## Abstract

Drug-induced immune haemolytic anaemia (DIIHA) is extremely rare. We herein report a case of DIIHA due to co-amoxiclav. A 53-year-old male being treated for left-sided pyelonephritis with intravenous co-amoxiclav developed symptoms and signs of anaemia on the third day of treatment. He was found to have evidence of haemolysis with a positive Coombs test and IgG antibodies and C3d. Co-amoxiclav was identified as the probable culprit, using the Naranjo adverse drug reaction probability scale. Upon discontinuation of the drug and blood transfusion, the patient's haematological parameters stabilised. Steroids were not required in the treatment. Diagnosis of DIIHA is made through a history of intake of co-amoxiclav, clinical and laboratory features of haemolysis, and a positive Coombs test. An autoantibody screen is most commonly negative. It is essential for clinicians to be aware about this rare complication caused by commonly prescribed drugs and be able to accurately diagnose and initiate treatment.

## 1. Introduction

Drug-induced immune haemolytic anaemia (DIIHA) is rare and occurs in about one per million per year [1]. The severity of DIIHA varies from acute severe intravascular haemolysis to subacute milder forms of extravascular haemolysis [2]. There are numerous drugs that have been incriminated in DIIHA. Common drugs causing DIIHA include antimicrobials (42%), nonsteroidal anti-inflammatory drugs (15%), and antineoplastic drugs (11%) [3]. Some of the common antibiotics causing DIIHA are cephalosporins, levofloxacin, nitrofurantoin, and piperacillin/tazobactam [4]. DIIHA is not commonly seen with co-amoxiclav, and we report a case of a DIIHA occurring in a 53-year-old male patient, prescribed this drug. Since DIIHA is generally under-reported, clinicians need to be aware about this rare but life-threatening adverse reaction to commonly prescribed drugs and its diagnosis and management.

## 2. Case Report

A 53-year-old male with a history of bronchial asthma (on inhaled salbutamol and corticosteroids) and diabetes mellitus (on gliclazide and metformin) presented to the National Hospital of Sri Lanka with fever, constitutional symptoms, dysuria, and left-sided loin pain for three days. Review of the other systems was normal. There were no known allergies to food or drugs. Patient informed about previous antibiotic usage as an outpatient; however, he was not sure which antibiotics were used and records were not traceable. However, he has not received intravenous antibiotics previously. On examination he was febrile, with significant left-sided renal angle tenderness, with normal vital signs, and no hepatosplenomegaly. The rest of the systemic examination was normal. He was empirically diagnosed with left-sided pyelonephritis and started on intravenous co-amoxiclav (1.2  g three times per day) on the day of presentation. Initial laboratory tests on admission showed a normal haemoglobin level of 13.7  g/dl (normal range 13–16.5  g/dL), a white blood cell count of 11,400/*μ*L (normal range 7,000–11,000/*μ*L), and a platelet count of 221,000/*μ*L (normal range 150,000–450,000/*μ*L). His urine microscopy showed a field full of pus cells, without any red cell or casts. Serum creatinine was normal (93 *μ*mol/l) and remained in the normal range throughout admission. Inflammatory markers were markedly elevated on admission (C-reactive protein 400  mg/dl). The initial clinical diagnosis was confirmed by ultrasound scan of the kidneys, which did not reveal any other abnormalities, including hepatosplenomegaly.

On day 3 of the admission, apart from mild fatigue, fever and other symptoms, including loin tenderness, have improved. However, physical examination revealed a pale conjunctivae and mucous membranes, along with mild icterus, but the rest of the systemic examination was normal. He did not complain of any bleeding manifestations, including melena, and digital rectal examination was normal. Repeat blood counts on day three revealed a reduction in the haemoglobin (9.5 g/dl) and C-reactive protein (190 mg/dl) levels. He had a reticulocyte production index of 4.6 (reticulocyte count 15%; haematocrit 30.0%), with indirect hyperbilirubinaemia (total bilirubin 17.6 *μ*mol/L; indirect bilirubin 11.4 *μ*mol/L), with normal hepatic transaminase levels. Changes in haemoglobin and red cell indices are summarized in Table 1. A review of the peripheral blood smear revealed polychromasia, red cell agglutination, and numerous nucleated red blood cells (RBCs), without evidence of bite cells or blister cells to indicate G6PD deficiency. Direct Coombs test was positive with IgG and C3d. Antibody screening was negative. His haemoglobin dropped to 4  g/dl on post-admission day 7 (reticulocyte production index 4.4%, haematocrit 13.2%). The following investigations for other causes of acquired haemolytic anaemia were negative: hepatitis B and C serology, retroviral screening, mycoplasma antibody, Ham test, EBV/CMV IgM antibodies, and anti-nuclear antigen (ANA).

In view of a possible DIIHA co-amoxiclav was changed to intravenous ciprofloxacin (400 mg twice daily). Additionally, 2 units of cross matched blood were transfused (blood group A positive). He reported improvement after the blood transfusion and discontinuation of co-amoxiclav. His haemoglobin gradually increased, and at day 6 after discontinuation of co-amoxiclav, it was 9.5 g/dL. The drug review showed that the only new drug prescribed to him was co-amoxiclav for the empirical treatment of pyelonephritis. Other drugs were unchanged and could not have accounted for the onset and subsequent improvement after discontinuation of co-amoxiclav. The patient had no further haemolytic episodes. The patient was doing well at the follow-up one month later. Using the Naranjo adverse drug reaction probability scale (our patient scored 7, which makes the drug reaction “probable”), the patient's diagnosis was confirmed as haemolytic anaemia, possibly caused by co-amoxiclav [5].

## 3. Discussion

Immune haemolytic anaemia occurs when IgG and/or IgM antibodies bind to the surface of the red cells, initiating cell destruction via the complement and reticuloendothelial systems. The condition is classified into either autoimmune, alloimmune, or drug-induced, depending on the antigenic stimulus responsible for the immune response [3]. The patient presented in this case report satisfies the diagnostic criteria for immune haemolytic anaemia, as he met the clinical and serological criteria of haemolysis (indirect hyperbilirubinaemia, decreasing haemoglobin level, and peripheral blood smear consistent with haemolysis), along with a positive Coombs test [6]. Co-amoxiclav is an antibiotic used to treat a number of bacterial infections, and it is a combination consisting of amoxicillin and beta-lactamase inhibitor, clavulanic acid. To our knowledge, DIIHA due to co-amoxiclav is only rarely described in the literature, although other beta-lactam antibiotics, including amoxicillin, are known to cause DIIHA [7, 8].

Several mechanisms causing DIIHA are described in the literature. In general, DIIHA can be mediated through drug-induced antibodies or through a mechanism called nonimmunologic protein adsorption [3]. Drug-induced antibodies can be either drug-dependent (DDA) or drug-independent antibodies (DIA) [3, 9]. DDAs need the presence of the drug to bind and lyse red cells, while DIAs can bind erythrocytes in absence of the causative drugs. However, DIIHA due to DIAs cannot be distinguished serologically from autoantibodies mediating warm autoimmune haemolytic anaemia, and hence, diagnosis depends upon clinical improvement on cessation of the causative drug [10]. The mechanism of co-amoxiclav-induced immune haemolysis resembles ceftriaxone induced haemolysis and is characterized by “immune complex-type” reactions [10]. Immune complex-type reactions occur with DDAs as a result of loose binding with red cells, while a covalent binding will result in a so-called “drug-adsorption mechanism” (penicillin-type) reaction [3, 9]. In cases of DDA-mediated DIIHA, the antibody screen is typically negative and only positive if tested in the presence of the drug or with RBC coated with the drug, respectively [10]. Although not performed in the current patient, this can be used as a test to confirm the diagnosis, where mixing washed group O RBCs, patient serum and the implicated drug will result in a strongly positive Coombs test. Subsequently, specificity can be demonstrated by showing that the Coombs test is negative when normal serum is used instead of patient serum and when drug is omitted from the reaction mixture.

Management of DIIHA includes the discontinuation of the suspected drug and red cell transfusion, resulting in a haemoglobin improvement over 7–10 days [3]. Our patient improved with these measures. There is no proven benefit and therefore no recommendation for steroid therapy in DIIHA, at least as far as drug-dependent antibodies are involved, and withdrawing the culprit drug is the most important step [10]. In cases of drug-independent antibodies, which are autoantibodies, steroid therapy can be tried, but also in these cases, the immediate withdrawal of the responsible drug is the most important therapeutic measure [10]. If glucocorticoids are required, they are recommended to be given for 1–3 weeks (1–2 mg/kg/day orally) [4, 5]. Intravenous steroids are reserved for those with severe haemolysis, requiring a rapid response, and other immunosuppressants such as cyclophosphamide and azathioprine can also be used in those who do not respond.

In conclusion, DIIHA is underestimated and under-reported, as many cases may not lead to dramatic haemolysis, requiring interventions. However, with increasing antibiotic use and increased reliance on newer generations of antibiotics, such rare but life-threatening adverse reactions may be more frequent. Therefore, it is essential for clinicians to recognize this rare complication caused by commonly prescribed drugs and be aware of its accurate diagnosis and management.

## Figures and Tables

**Table 1:** Changes in haemoglobin and red cell indices.

	Days				
	Baseline	3	7 (medication stopped)	10 (2 days after stopping)	14 (6 days after stopping)
Number of red cells (x10^9^/L)	4.61	3.43	1.24	1.98	3.20
Haemoglobin (g/dL)	13.7	9.5	4	6	9.6
Haematocrit (HCT) (%)	39.4	30.8	13.2	19.7	29.7
Mean corpuscular volume (MCV) (fL)	85.4	89.8	106.4	99.5	92.8
Mean corpuscular haemoglobin (MCH) (pg)	29.7	27.7	32.2	30.3	30
Mean corpuscular haemoglobin concentration (MCHC) (g/dL)	34.7	30.8	30.3	30.4	32.3
Red cell distribution width (RDW) (%)	12.7	14.7	17.7	24.2	17.8
Reticulocyte count	1.5%	5.9%	15%	11.6%	5.4%
Reticulocyte index	1.3%	3.9%	4.4%	5.1%	3.6%
